# Advances in clinical applications of kisspeptin-GnRH pathway in female reproduction

**DOI:** 10.1186/s12958-022-00953-y

**Published:** 2022-05-23

**Authors:** Kai-Lun Hu, Zimiao Chen, Xiaoxue Li, Enci Cai, Haiyan Yang, Yi Chen, Congying Wang, Liping Ju, Wenhai Deng, Liangshan Mu

**Affiliations:** 1 Center for Reproductive Medicine, Peking University Third Hospital, No.49 Huayuan North Road, Haidian District, Beijing, People’s Republic of China 100191; 2 Zhejiang MedicalTech Therapeutics Company, No.665 Yumeng Road, Wenzhou, People’s Republic of China 325200; 3 Department of Endocrinology, The First Affiliated Hospital of Wenzhou Medical University, Wenzhou, People’s Republic of China 325000; 4 Department of Nutrition and Food Science, College of Food, Agricultural and Natural Resource Sciences, University of Minnesota, Twin Cities, Minneapolis, MN 55455 USA; 5 Reproductive Medicine Center, The First Affiliated Hospital of Wenzhou Medical University, Wenzhou, People’s Republic of China 325000; 6 Key Laboratory of Laboratory Medicine, Ministry of Education, School of Laboratory Medicine and Life Sciences, Wenzhou Medical University, Wenzhou, People’s Republic of China 325006

**Keywords:** Kisspeptin, KISS1R, Hypothalamus, Hypothalamic-pituitary-ovarian axis, Female reproduction

## Abstract

**Background:**

Kisspeptin is the leading upstream regulator of pulsatile and surge Gonadotrophin-Releasing Hormone secretion (GnRH) in the hypothalamus, which acts as the key governor of the hypothalamic-pituitary-ovary axis.

**Main text:**

Exogenous kisspeptin or its receptor agonist can stimulate GnRH release and subsequent physiological gonadotropin secretion in humans. Based on the role of kisspeptin in the hypothalamus, a broad application of kisspeptin and its receptor agonist has been recently uncovered in humans, including central control of ovulation, oocyte maturation (particularly in women at a high risk of ovarian hyperstimulation syndrome), test for GnRH neuronal function, and gatekeepers of puberty onset. In addition, the kisspeptin analogs, such as TAK-448, showed promising agonistic activity in healthy women as well as in women with hypothalamic amenorrhoea or polycystic ovary syndrome.

**Conclusion:**

More clinical trials should focus on the therapeutic effect of kisspeptin, its receptor agonist and antagonist in women with reproductive disorders, such as hypothalamic amenorrhoea, polycystic ovary syndrome, and endometriosis.

## Background

The normal function of the female reproductive system relies on the coordination of the hypothalamic-pituitary-ovary (HPO) axis [1]. HPO axis is governed by the Gonadotrophin-Releasing Hormone (GnRH) from the hypothalamus and subsequent follicle-stimulating hormone (FSH) and luteinizing hormone (LH) from the pituitary [2]. The pulsatile secretion of these hormones, which is induced by the negative feedback of sex steroids, is required for ovarian maturity and cyclic function at puberty and in adulthood [3]. In addition to the pulsatile secretion of GnRH and gonadotrophins (FSH and LH), the surge mode of GnRH release and subsequent LH surge is induced by the positive feedback of serum sex steroids during the periovulatory stage, which is required for triggering ovulation in female mammals [4]. However, the mechanisms underlying the negative and positive feedback effects of sex steroids remain uncharacterized until recent years. Although GnRH plays a fundamental role in the female reproductive system, the principle receptor (estrogen receptor α, ERα) mediating both negative and positive sex steroids feedback processes in the hypothalamus is not detected in GnRH neurons [5]. Studies related to kisspeptin, the main upstream regulator of pulsatile and surge secretion of GnRH, have been of considerable interest in the past decade. Kisspeptin neurons are detected in two hypothalamic regions, including the anteroventral periventricular nucleus (AVPV) and arcuate nucleus (ARC), which mediate the positive and negative feedback of sex steroids, respectively [6, 7]. In many mammals, including humans, kisspeptin stimulates GnRH release by binding to the kisspeptin receptor (KISS1R) in GnRH neurons. Homogeneous mutation of either KISS1 or KISS1R is associated with hypogonadotropic hypogonadism and infertility [8–14]. Being the main upstream regulator of pulsatile and surge GnRH release, plentiful applications of kisspeptin or its analogs in female reproduction have been identified in recent years. This review aimed to summarize the emerging evidence of clinical applications of kisspeptin and its analogs in the female reproductive system.

### A short review of kisspeptin gene, peptide, and its receptor

**Fig. 1:**
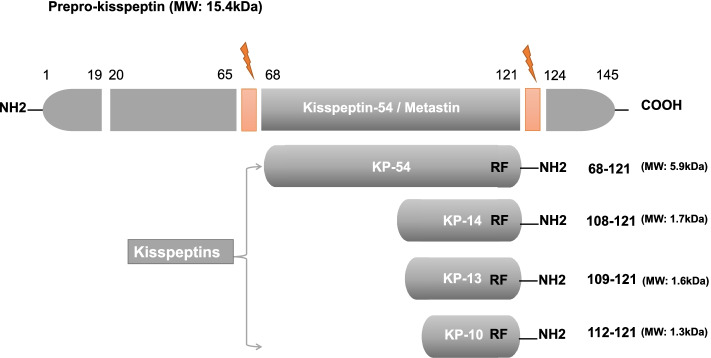
The structure of kisspeptins in humans. Kisspeptins are derived from a 145-amino-acid prepro-kisspeptin (encoded by KISS1 gene). The cleavage sites at 68 and 121 of prepro-kisspeptin lead to the production of the RF-amidated KP54. Shorter kisspeptins (KP-10, − 13, and − 14) share a common C terminus and RF-amidated motif with KP54. Modified from [12, 17]

Kisspeptin was initially identified as a human metastasis suppressor of malignant melanoma in 1996 [15]. In humans, kisspeptin is encoded by the KISS1 gene, which is located on the long (q) arm of chromosome 1 at q32 [16]. At first, KISS1 produces an unstable and biologically inactive prepro-kisspeptin which consists of 145-amino-acid. Then four biologically active peptides are generated after the cleavage of prepro-kisspeptin within the cell: kisspeptin-54 (KP54), kisspeptin-14 (KP14), kisspeptin-13 (KP13), kisspeptin-10 (KP10) [9]. These peptides have a preserved C-terminal region that contains an Arg-Phe-NH2 (RF-NH2) motif, which enables the high-affinity binding to and full activation of KISS1R (Fig. 1). KP54 has been considered the main product of the KISS1 gene in humans, and it can be further cleaved into KP14, KP13, and KP10.

**Fig. 2:**
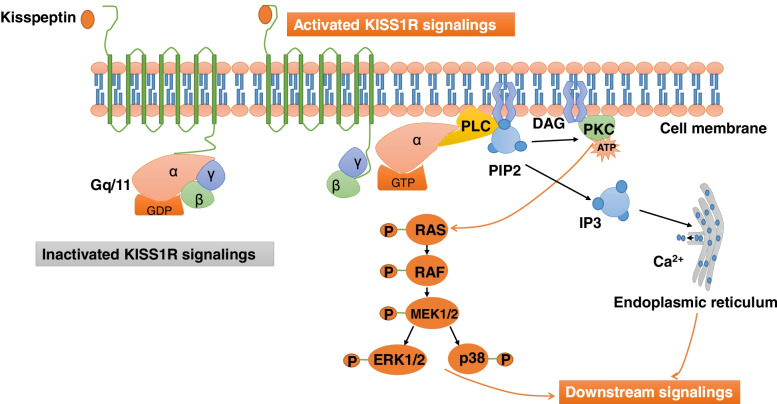
Kisspeptin/KISS1R signalings. When kisspeptin binds to the seven-transmembrane domain receptor, KISS1R, the intracellular portion of KISS1R leads to the phosphorylation of Gq/11. The α-subunit of Gq/11 then activates PLC, which subsequently cleaves PIP2 into IP3 and DAG. IP3 promotes intracellular Ca2+ release from the endoplasmic reticulum, while DAG promotes the phosphorylation of PKC which further induces the phosphorylation of ERK1/2 and p38. DAG, diacylglycerol; ERK1/2, extracellular signal-regulated kinase; IP3, inositol 1,4,5-triphosphate; PI3K, phosphatidylinositol-3-kinase; PIP2, phosphatidylinositol 4,5-bisphosphate; PKC, protein kinase C; PLC, phospholipase C. Modified from [17]

KISS1R belongs to the family of the seven-transmembrane G-protein-coupled receptor, which binds extracellular substances and transmits signals to an intracellular molecule [18]. Research first identified the physiological role of kisspeptin and KISS1R in the HPO axis in 2003, which therefore revolutionized the field of the neuroendocrine–reproductive system [13, 14]. As with many GPCRs, activation of the kisspeptin/KISS1R signaling pathway further activates phospholipase C (PLC) through a Gαq mediated pathway and subsequently promotes the production of diacylglycerol (DAG), inositol- [1, 4, 5]-triphosphate (IP3). DAG, in turn, promotes the phosphorylation of protein kinase C (PKC), which then initiates the mitogen-activated protein kinase (MAPK) and extracellular signal-regulated kinase (ERK) signaling pathway, while IP3 increases intracellular calcium release to the cytoplasm [12, 19] (Fig. 2).

### Distribution of kisspeptin and KISS1R in the hypothalamus

The normal function of the adult female HPO axis critically depends on appropriate secretory patterns of GnRH. However, the GnRH neurons are devoid of estrogen receptor α, which is responsible for both negative and positive feedback in the female HPO axis [20]. The intermediary neurons mediating gonadal feedback remained unknown until the 2000s [13, 14]. Mutations in the KISS1 gene or KISS1R can lead to idiopathic hypogonadotropic hypogonadism and delay pubertal maturation of the gonadotropic axis, suggesting that KISS1R is indispensable for normal GnRH secretion [10, 11, 13, 14, 21–25].

In the murine hypothalamus, kisspeptin mRNA and protein have been detected with dense concentrations in the nucleus involved in regulating GnRH and gonadotropin secretion, including the AVPV, the periventricular nucleus (PeN), as well as the ARC [26, 27]. Additionally, kisspeptin mRNA is present in a few cells in the anterodorsal preoptic nucleus, the medial amygdala, and the bed nucleus of the stria terminals [26, 27]. Despite the similar overall distribution of neurons that express kisspeptin between adult males and females in the murine, a significant sex difference in the number of kisspeptin neurons in the AVPV and PeN area has been found, with much larger numbers of kisspeptin neurons in the female than the male [27–29]. Due to ethical considerations, investigations on the expression kisspeptin expression and KISS1R in the hypothalamus of humans are often limited or precluded. Nevertheless, studies performed in nonhuman primates can provide some global insight into the anatomy and physiology of kisspeptin and KISS1R in the hypothalamus in humans. In the male rhesus monkey, expression of kisspeptin has been found in the anterior ARC and the internal zone of the median eminence (ME), but not in the preoptic area (POA) or the AVPV [30, 31]. While in the female rhesus monkey, kisspeptin-expressing cells were detected in the POA and ARC, and the expression of kisspeptin was higher in the late follicular phase of the menstrual cycle than in the luteal phase [32]. Initial studies investigating the neuroanatomical distribution of kisspeptin neurons in the human hypothalamus were carried out in autopsy samples. These studies demonstrated that kisspeptin neurons were located predominantly in the infundibular nucleus (which is the homolog of the ARC in rodents) and the rostral POA, with no kisspeptin neurons in the rostral periventricular region of the third ventricle [33]. Likewise, the sexual dimorphism in the distribution of kisspeptin neurons is found in the human hypothalamus, with more kisspeptin cell bodies in the infundibular nucleus in women than in men [34].

The expression of KISS1R in the brain was not identified until 1999 [18]. Using Northern blot and in situ hybridization, Lee et al. demonstrated that KISS1R was expressed in the pons, midbrain, thalamus, hypothalamus, hippocampus, amygdala, cortex, frontal cortex, and striatum in rats. A few years later, Irwig et al. revealed a more intricate expression of KISS1R in the forebrain of rats, including the medial septum, medial preoptic area, lateral preoptic area, median preoptic nucleus, anterior hypothalamus, and lateral hypothalamus [35]. Furthermore, studies using double-label in situ hybridization to investigate whether KISS1R was expressed in GnRH neurons found that more than 75% of GnRH neurons in the ME and POA regions coexpress KISS1R and express high levels of c-fos in response to kisspeptin-10 [35, 36]. These studies suggest that kisspeptin acts directly on the GnRH neurons to regulate the HPO axis.

### Kisspeptin mediates steroid feedbacks on the HPO axis

**Fig. 3:**
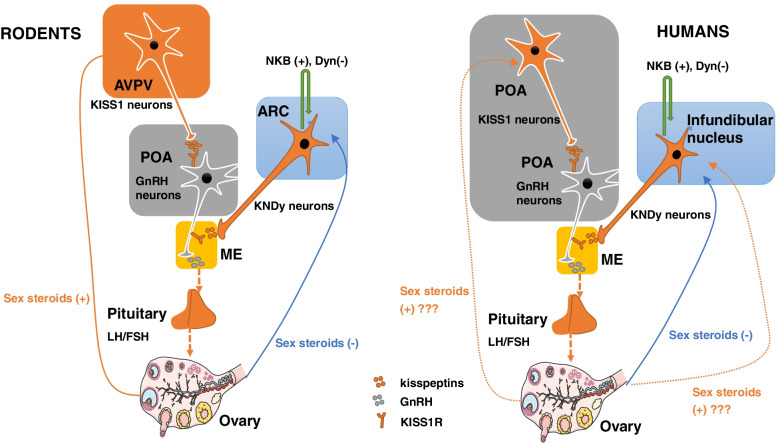
Schematic diagram showing how kisspeptin regulates hypothalamus-pituitary-ovary axis in rodents and humans. In rodents, KISS1 neurons within anteroventral periventricular nucleus (AVPV) and the arcuate nucleus (ARC) are responsible for the positive feedback (red) and negative feedback (blue) of sex steroids, respectively. While in humans, KISS1 neurons within the infundibular nucleus are responsible for the negative feedback (blue) of sex steroids. The area where KISS1 neurons mediate the positive feedback (red) is unclear. KISS1 neurons in the infundibular (humans)/arcuate (rodents) nucleus co-express neurokinin B (NKB) and dynorphin (Dyn), and are therefore named KNDy neurons. NKB and Dyn autosynaptically regulate pulsatile kisspeptin secretion in KNDy neurons, with NKB being stimulatory and Dyn inhibitory. POA, preoptic area; ME, median eminence

There are two different modes of GnRH/LH secretion in females: the pulsatile and the surge modes [37]. The pulsatile GnRH/LH secretion, which is more predominant throughout the menstrual cycle, promotes the maturation of ovarian follicles and sex hormone production in the ovary. The process is controlled by the negative sex steroid feedback actions on the kisspeptin neurons in the ARC area in various mammals or its equivalent infundibular area in primates (including humans) [38]. Kisspeptin neurons in the ARC express ERα and are tonically suppressed by estrogen signals, providing a plausible pathway for transmitting the negative feedback actions of sex steroids on GnRH [38, 39]. Attributed to the kisspeptin neurons in the ARC co-express neurokinin B and dynorphin neurons, the term KNDy (Kiss1/NKB/Dyn) neurons are used to identify these cells [40–42]. Functional analyses conducted in animal models lead to the proposal of an oscillatory network within KNDy neurons, where NKB and Dyn, in an auto/paracrine manner, regulate the output of kisspeptin onto GnRH neurons in an opposite way. Thus, NKB stimulates kisspeptin release via its receptor NK3R in the KNDy neurons, therefore inducing GnRH secretion from the median eminence of the hypothalamus and subsequent pulsatile release LH from the pituitary. In contrast, Dyn seemingly operates as an inhibitory role in kisspeptin secretion, thereby GnRH/LH pulsatility [43–46] (Fig. 3). A previous study showed that inactivating mutations of the genes encoding NKB or its receptor (NK3R) in humans led to a state of central hypogonadism similar to that of the inactivating mutations of the kisspeptin pathway [47], indicating that the NKB signal is indispensable for kisspeptin secretion in KNDy neurons. Moreover, continuous KP10 infusion can restore GnRH/LH pulsatility in patients (men and women) with loss-of-function mutations in NKB or NK3R, suggesting that kisspeptin is able to stimulate pulsatile GnRH/LH secretion in the absence of NKB signalings in humans [48]. This result is further confirmed by the unaffected LH increase induced by kisspeptin-10 in the presence of NK3R antagonist in healthy men [49] (Table 1).

**Table 1:** Kisspeptin and its analogs regulate LH secretion in women.

Studies	Agonists	Participants	Treatment	Baseline LH (IU/L)	Max LH (IU/L) after treatment
(Dhillo et al. 2007) [50]	KP54	26-32 healthy women	sc bolus 0.2, 0.4, 0.8, 1.6, 3.2, and 6.4 nmol/kg in the follicular phase; sc bolus 0.4 nmol/kg in each phase of the menstrual cycle (follicular, preovulatory, and luteal phase)	follicular: 4.2 preovulatory: 14.5 luteal: 3.6 in each phase	The highest increase: preovulatory phase
(Jayasena et al. 2011) [51]	KP10	35 healthy women	iv bolus 0.3, 1.0, 3.0, or 10 nmol/kg in follicular phase and preovulatory phase women	2.9 3.8/6.8	Increase only in preovulatory phase women
(Jayasena, Comninos, Veldhuis, et al. 2013) [52]	KP54	6 healthy women	sc bolus 0.15, 0.30, 0.60 nmol/kg	1.2-9.0	Increase: 2.3 in 0.60 nmol/kg
(Jayasena, Comninos, Nijher, et al. 2013) [53]	KP54	5 healthy women	twice-daily sc injections 6.4 nmol/kg during menstrual days 7-14	5.7	Increase: 8.6 on day 7 Increase: 12.7 on day 14
(Narayanaswamy et al. 2016) [54]	KP54	4 healthy women	sc infusion 0.1, 0.3 or 1.0 nmol/kg/h for 8 h	4.6	Around 5, 10, 10
(Skorupskaite et al. 2016) [55]	KP10	20 healthy women	iv infusion 4 μg/kg/h for 7 h	4.8-5.6	21.6
(Skorupskaite et al. 2018) [56]	KP10	Postmenopausal women (n=11)	iv bolus 0.3 μg/kg	Unclear	No increase
(Chan et al. 2012) [57]	KP10	10 women in early follicular phase, 3 in preovulatory phase, and 14 in midluteal phase	iv bolus 0.24 nmol/kg	follicular phase: 2.9 preovulatory phase: 34.7 midluteal phase: 3.4	follicular phase: 3.8 preovulatory phase: 61.3 midluteal phase: 7.1
(George, Anderson, and Millar 2012) [58]	KP10	10 women in early follicular phase, 6 post-menopausal, 4 with progestogen implants, 4 with COCP	iv bolus 0.3 μg/kg	follicular phase: 6.3, post-menopausal: 35.3, progestogen implants: 4.6, CPCP: 2.3	follicular phase: 9.4, post-menopausal: 44.7, progestogen implants: 7.5, CPCP: 3.7
(Jayasena, Abbara, Comninos, et al. 2014) [59]	KP54	53 women undergoing IVF (preovulatory phase)	sc bolus 1.6 nmol/kg, 3.2 nmol/kg, 6.4 nmol/kg, 12.8 nmol/kg	0.8, 1.4, 1.3, 1.3	6.6, 3.8, 7.9, 8.8
(Abbara et al. 2017) [60]	KP54	62 women at high risk of OHSS (preovulatory phase)	sc bolus 9.6 nmol/kg second sc bolus 9.6 nmol/kg 10 h later	Around 2 Around 20	Around 50 Around 24
(Romero-Ruiz et al. 2019) [61]	KP54	12 women with PCOS	sc 9.6 nmol/kg twice daily for 21 days	10.8	7-day after completion of treatments: 13.4
(Skorupskaite et al. 2020) [62]	KP10	10 women with PCOS	iv infusion 4 μg/kg/h for 7 h	5.2	7.8
(Jayasena et al. 2009) [63]	KP54	10 women with HA	sc 6.4 nmol/kg twice daily for two weeks	2.6	24.0 at 4 h Increase: 2.5 on day 14
(Jayasena et al. 2010) [64]	KP54	20 women with HA	sc 6.4 nmol/kg twice daily for two weeks sc 6.4 nmol/kg twice weekly for eight weeks	2.5 1.8	23.3 at 6 h; 3.5 on day 14 1.9 after eight weeks
(Jayasena, Abbara, Veldhuis, et al. 2014) [65]	KP54	5 women with HA	iv infusion 1.00 nmol/kg/h for 8 h	1.3	15.4
(Millar et al. 2017) [66]	KP10	2 women with HA	iv infusion 1.5 μg/kg/h for 12 h	Patient 1: 5.3 Patient 2: 1.2	Patient 1: 25.4 Patient 2: 5.2
(Abbara et al. 2020) [67]	KP54 TAK-448	9 healthy women, 6 women with PCOS and 6 with HA	TAK-448 (doses 0.01 and 0.03 nmol/kg), KP54 (9.6 nmol/kg)	Healthy: 3.7 PCOS: 4.4 HA: 2.9	Amplitude of LH: similar after KP54 and MVT-602 Timing of peak LH: much later after MVT-602

Abbreviations: *IVF* in vitro fertilization, *OHSS* Ovarian Hyperstimulation Syndrome, *HA* hypothalamic amenorrhea, *PC* prostate cancer, *PCOS* polycystic ovary syndrome, *TSH* thyroid-stimulating hormone, *sc* subcutaneous, *iv* intravenous, *COCP* combined oral contraceptive pills.

The surge mode of GnRH/LH secretion occurs periodically at the mid-cycle (preovulatory stage) in adult females [68]. Generation of this preovulatory surge critically relies on a switch from the predominant negative feedback to the positive feedback of estrogen. The high concentrations of circulating estradiol derived from the dominant ovarian follicles induce stimulatory signals to GnRH neurons to increase, rather than suppress, GnRH secretion [68]. The first evidence for a putative role of kisspeptin neurons in estrogen positive feedback came from rodent studies, which demonstrated kisspeptin expression in the AVPV area was reduced after ovariectomy and increased after estrogen replacement [6]. Kisspeptin mRNA levels in the AVPV area increased during the preovulatory surge among female rats. In comparison, immunoneutralization of central kisspeptin or selective blockade of kisspeptin actions with KISS1R antagonists blocked the preovulatory LH surge in cyclic rats [69, 70]. Collectively, these studies suggest the involvement of kisspeptin neurons in the AVPV area in mediating the positive feedback of sex steroids on GnRH/LH secretion during the preovulatory period [68] (Fig. 3). In humans, there is no functional evidence for the anatomical region of kisspeptin neurons that mediate the positive estrogen feedback to induce the GnRH/LH surge. Furthermore, there has no homologous area in humans to the AVPV nucleus in rodents. Because kisspeptin neurons are located predominantly in the infundibular nucleus (which is the homolog of the ARC in rodents) and the rostral POA in the human hypothalamus [26, 33, 71], kisspeptin neurons in one of the two areas may be responsible for the positive sex steroids feedback (Fig. 3).

### Kisspeptin peptides and related analogs

#### Native Kisspeptin peptides

In humans, all native kisspeptins (KP54, KP14, KP13, and KP10) share a typical C-terminal decapeptide sequence, enabling these peptides to bind to and activate KISS1R [9, 72]. Therefore, KP10 and KP54 are most commonly investigated in humans. While the reported half-life of KP54 varies from 28 min to 1.8 h in humans, it is clear that KP54 has a longer terminal half-life than KP10 in humans [73–75]. Although Kotani et al. demonstrated that the bioactivity of KP54 and KP10 are equipotent and the two peptides bind to KISS1R with a similar affinity in humans [72, 76], KP54 is more suitable for bolus administration than KP10 in humans due to their pharmacokinetic properties. A bolus administration of KP54 can lead to a modest rise of LH levels at the follicular phase in healthy women, which does not happen after a bolus administration of KP10 [51] (Table 1). Additionally, the maximum LH rise is much more significant after a bolus administration of KP54 than after a similar dose of KP10 in healthy men [74, 77]. Their blood-brain barrier permeability explains the differentiation in biological activity in vivo between KP10 and KP54. Peripheral administration of KP54 can activate c-FOS in GnRH neurons behind the blood-brain barrier (BBB), but this is not seen after peripheral administration of KP10 [73]. Nevertheless, both KP54 and KP10 can elicit an endocrine response by activating kisspeptin receptors on GnRH neurons in the median eminence area, which is external to the BBB.

#### Kisspeptin receptor agonists

**Fig. 4:**
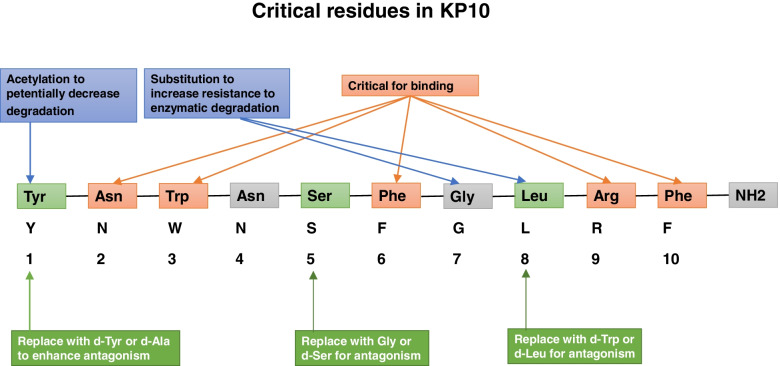
Critical residues in KP10. Residues highlighted with red are important for KISS1R binding; Residues highlighted with blue are important for KISS1R activation. Modified from [84]

While KP54 is more stable and has a longer duration of action in vivo, KP10 has a shorter amino acid sequence and, therefore, is less expensive for manufacturers to produce analogs [73–75]. Numerous kisspeptin analogs have been synthesized, including those containing substitutions with unnatural amino acids [78–82]. Kisspeptins are susceptible to enzymatic proteolysis by matrix metalloproteinases (MMPs) at their Gly-Leu peptide bond in the C-terminal region [79, 83] (Fig. 4). Therefore, substituting these dipeptides may afford resistance to enzymatic degradation and maintain the KISS1R agonistic activity, such as (E)-Alkene- and hydroxyethylene-type isostere-containing analogs [78]. In another study, researchers designed and synthesized many KP10 analogs, among which the substitution of arginine at position 53, N(ω)(−)methylarginine analog 8 shows 3-fold more potent bioactivity than KP10, with trypsin cleavage resistance between positions 53 and 54 [85]. However, not all kisspeptin receptor agonists can maintain the in vivo efficacy with an increased in vitro stability. FTM080, a kisspeptin receptor agonist with a half-life of 6.6 hours in murine serum, is less effective in maintaining the duration of LH secretion in ewes than native KP10 [86]. Another KP10 analog, designed by the substitution of D-tyrosine for a tyrosine residue at position 1 ([dY](1)KP-10), binds to KISS1R with lower affinity than KP-10 and exhibits comparable bioactivity in vitro [87]. Interestingly, peripheral administration of [dY](1)KP-10 can increase plasma LH and testosterone more potently than KP-10 at 20 min postinjection in mice, indicating the affinity of kisspeptin analogs in vitro is not necessarily associated with their potency in vivo [87]. (Table 2).

**Table 2:** Characteristics of kisspeptin receptor agonists

Kisspeptin receptor agonists	Sequence	Affinity	Half-life (t½)	Potency (in vivo and in vitro)	BBB permeability	Related studies
KP10	H-Tyr-Asn-Trp-Asn-Ser-Phe-Gly-Leu-Arg-Phe-NH_2_	Reference	55 s to 4 min in humans and 34 s in mice	Reference	NO	(Beltramo and Decourt 2018; d'Anglemont de Tassigny et al. 2017 [73]; Jayasena et al. 2011 [51])
KP54	H-Gly-Thr-Ser···Tyr-Asn-Trp-Asn-Ser-Phe-Gly-Leu-Arg-Phe-NH_2_	Comparable	1.3–1.8 h in humans and 32 min in mice (28 min in humans in one study (Dhillo et al. 2005))	Comparable in vitro, higher in vivo	YES	(George et al. 2011 [77]; Abbara et al. 2020 [67]; d'Anglemont de Tassigny et al. 2017 [73]; Beltramo and Decourt 2018; Abbara, Clarke, and Dhillo 2021 [88])
FTM080	4-fluorobenzoyl-Phe-Gly-Leu-Arg-Trp-NH2	Comparable	6.6 h in murine serum	Comparable in vitro and lower in vivo	Maybe yes (it induces ovulation in musk shrews)	(Whitlock et al. 2015 [86]; Inoue et al. 2011)
[dY](1)KP-10	H-d-Tyr-Asn-Trp-Asn-Ser-Phe-Gly-Leu-Arg-Phe-NH_2_	Lower in vitro	Unclear	Comparable in vitro and higher in vivo	Unclear	(Curtis et al. 2010 [87])
Compound 6	palm-γ-Glutamyl-Tyr-Asn-Trp-Asn-Ser-GlyΨ[Tz]Leu-Arg(Me)-Tyr-NH2	Higher	Longer than KP10	Higher in vitro and in vivo	Probably Yes	(Decourt et al. 2016 [89]; Decourt et al. 2019)
Compound 26 (KISS1-305)	H- d-Tyr-d-Pya(4)-Asn-Ser-Phe-azaGly-Leu-Arg(Me)-Phe-NH2	Comparable	6.76 min in rats	Higher in vitro	Probably Yes	(Asami et al. 2013 [90]; Matsui et al. 2012 [91])
TAK-448 (MVT-602)	Ac-d-Tyr-Hydroxyproline (Hyp)-Asn-Thr-Phe-azaGly-Leu-Arg(Me)-Trp-NH 2	Comparable	1.6-2.2 h in humans	Comparable in vitro and higher in vivo	YES	(Yoshida et al. 2012; Matsui et al. 2014 [92]; Abbara et al. 2020 [67])
TAK-683	Ac-d-Tyr-d-Trp-Asn-Thr-Phe-azaGly-Leu-Arg(Me)-Trp-NH2	Comparable	1.18-17.94 h in humans	Comparable in vitro and higher in vivo	Unclear	(Yoshida et al. 2012; Matsui et al. 2014 [92]; Scott et al. 2013 [93]; Abbara et al. 2020 [67]; Rahayu et al. 2017)
RF9	1-adamantane carbonyl-Arg-Phe-NH2	Higher	40 min in mare circulation	Lower in vitro	Unclear	(Liu and Herbison 2014 [94]; Min et al. 2015 [95]; Korthanke et al. 2017)
Bz-Arg-Trp-NH2	Bz-Arg-Trp-NH2	Higher	121 min in rat serum	Lower in vitro and comparable in vivo	Unclear	(Doebelin et al. 2016 [96])

KP10 analog C6 is designed by inserting an albumin-binding motif in the isoGlutamyl on the N-terminal amine to protect it from fast excretion and ω-methylation of arginine at position 9 to improve resistance to proteolytic degradation without affecting potency [89]. Additionally, the insertion of triazole between the leucine and the glycine may increase the lipophilicity and, therefore, membrane permeability of C6 [89]. Thus, C6 has a longer half-life in vivo than KP10 due to increased proteolytic stability [97]. Additionally, C6 is more potent than KP10, as demonstrated in ewes [89]. Electrophysiological recordings of GnRH neurons from murine brain slices demonstrate that C6 exerts a direct stimulatory action on GnRH neurons in the hypothalamus and induces ovulation, indicating a possible increase in lipophilicity of this molecule compared to KP10, at least in mice [89].

Compound 26 (C26) is a stable nonapeptide designed by the N-terminal truncation of KP10, and it has KISS1R binding affinities comparable to KP10 with improved serum stability [90, 91]. Continuous administration of C26 significantly suppresses testosterone in male rats, and the plasma testosterone levels following C26 treatment decreas faster than those receiving KP10 [90]. In addition, C26 can induce ovulation in female rats, indicating a possible increase in lipophilicity of this molecule compared to KP10, at least in rats [90].

TAK-448 and TAK 683 are two kisspeptin analogs designed bymodifying KP10 with nine amino acids [90, 98]. Compared with KP10, the two analogs show comparable KISS1R-binding affinity and potency with increased water solubility and half-life in vivo [92]. The two analogs are originally developed for clinical use in patients with prostate cancer due to kisspeptin’s ability to induce tachyphylaxis when given chronically at high doses [99, 100]. Inducing castrate levels of testosterone in vivo is beneficial intreating prostate cancer, in addition to kisspeptin’s putative antimetastatic activity [72]. A recent study evaluated the potency of TAK-448 (also known as MVT-602) to induce LH rise in the follicular phase of healthy women [67]. Despite the comparable half-life between TAK-448 and KP54, TAK-448 induces a prolonged duration of LH rise, though to a similar amplitude as KP54 [67], indicating that TAK-448 has increased resistance to degradation by proteosomes and can induce signals for a longer duration. In vitro experiments demonstrates that TAK-448 is more potent and induces a longer duration of GnRH neuronal firing than KP54 (115 vs. 55 min) [67].

Using alanine scanning and amino acid substitution, researchers found that Phe6, Arg9, and Phe10 residues lie on one face and constitute a binding pharmacophore of KP10, which is essential to the receptor binding [101]. Small molecules mimicking vital elements of this pharmacophore site can bind to and activate the kisspeptin receptor, albeit with reduced potency compared to KP10 [101]. RF9 (1-adamantane carbonyl-Arg-Phe-NH2) is an antagonist of receptors for the mammalian gonadotropin-inhibitory hormone receptor [102, 103]. Central administration of RF9 can induce a dose-dependent increase of gonadotropin levels in adult male and female rats and augment the gonadotropin-releasing effects of kisspeptin [102]. Interestingly, recent studies demonstrated that the stimulatory action of RF9 on gonadotropin release is mediated via direct KISS1R agonism, though the potency is slightly lower than KP10 in vitro [94, 95]. Additionally, RF9 can exert a dose-dependent excitatory action on brain slices of GnRH neurons in POA in adult male and diestrous female mice [94]. Another small molecule, benzoylated dipeptide Bz-Arg-Trp-NH2, shows promising KISS1R agonistic activity and better stability than the endogenous kisspeptins in rats. However, its selectivity towards other RFamide receptors such as NPFF1R and NPFF2R needs to be resolved [96]. Because the cost of synthesis and the stability of small molecules are more favorable than those of kisspeptin, it is worth investigating whether these small molecules can facilitate oral administration in the future and provide a therapeutic advantage in clinical trials. Additionally, more synthetic work is required to improve their selectivity toward KISS1R.

#### Kisspeptin receptor antagonists

In addition to Phe6, Arg9, and Phe10, the amino acids residues Asn2 and Trp3 are also critical for receptor binding, and Tyr1 and Leu8 are necessary for receptor activation [84, 101, 104] (Fig. 4). Kisspeptin antagonism, P234, results from the substitution of Leu8 with d-Trp and of Ser5 with Gly, with further enhancement by the substitution of Tyr 1 with D-Ala [105]. P234 can bind to the kisspeptin receptor but can not induce signalings, which can induce around 90% inhibition of KP10 activated signalings in KISS1R-transfected CHO cells [105] (Table 3). While P234 inhibits kisspeptin-induced stimulation of GnRH/LH, the basal GnRH/LH levels are not affected by P234 [105]. P271 is a modified version of P234, with a penetrating sequence attached to enhance BBB permeability. P271 can inhibit the GnRH/LH surge induced by kisspeptin when administered systemically in rats and ewes [70, 106, 107]. Small molecule antagonists for KISS1R have also been synthesized via a 2-acylamino-4,6-diphenylpyridine scaffold [108]. Compound 15a, a minor molecule KISS1R antagonist, modified from compound 91, is designed with further optimization by containing a piperazine ring that enhances BBB permeability [109]. Compound 15a exhibits similar binding affinity and lower inhibition ability for KISS1R in vitro and in vivo in male rats compared to P234 [109]. Unlike peptide 234, which needs to be given by injection, compound 15a is a small molecule antagonist that is likely to facilitate oral administrationand is more applicable for clinical trials.

**Table 3:** Characteristics of kisspeptin receptor antagonists

Kisspeptin receptor antagonists	Sequence	Affinity	Half-life (t½)	Potency (in vivo and in vitro)	BBB permeability	Related studies
P234	(D-Ala)-Asn-Trp-Asn-Gly-Phe-Gly-(D-Trp)-Arg-Phe-NH2	Reference	Unclear	90% inhibition of KP10 activated signalings	Unclear	(Roseweir et al. 2009 [9]; Pineda et al. 2010 [70]; Albers-Wolthers et al. 2017 [106])
P271	Arg-Arg-Met-Lys-Trp-Lys-Lys-Tyr-(D-Ala)-Asn-Trp-Asn-Gly-Phe-Gly-(D-Trp)-Arg-Phe-NH2	Comparable with P234	Unclear	Comparable with P234	Probably Yes	(Pineda et al. 2010 [70]; Smith et al. 2011 [107]; Albers-Wolthers et al. 2017 [106])
Compound 15a	2-Acylamino-4,6-diphenylpyridine	Comparable with P234	Unclear	Lower than P234	Probably Yes	(Kobayashi et al. 2010 [108, 109])

### Clinical applications of kisspeptin in female reproductive health

The safety of kisspeptin and its specific role in regulating GnRH/LH secretion in the hypothalamus promote its application in clinical trials. Up to date, no increased adverse events (such as nausea or changes in heart rate and blood pressure) of kisspeptin and related analogs have been found in clinical trials when compared to the placebo, and no serious adverse events related to kisspeptin or related analogs have been reported [50, 75, 93, 99]. Additionally, while kisspeptin stimulates GnRH/LH secretion in the hypothalamus, it has no significant effect on the secretion of growth hormone, prolactin, and thyroid-stimulating hormone [110]. Therefore, there is an expansive application of kisspeptin based on its hypothalamic role, including female and male contraception, induction of ovulation for IVF, delayed puberty, precocious puberty, and hypothalamic amenorrhoea [84, 88]. Currently, only KP54, KP10, TAK-683, and TAK-448 have been taken forward into clinical trials in humans [93, 99] (Table 1). Moreover, KP54 is more commonly used in human trials than KP 10 due to its ability to cross the BBB and its longer half-life in vivo [73].

#### Trials in healthy women at reproductive age

Dhillo et al. (2007) investigated the effects of KP54 intravenous administration in healthy women and found that the elevation of plasma KP54 levels significantly increased circulating LH, FSH, and testosterone levels [75]. Two years later, the same research group investigated the effects of a subcutaneous bolus injection of different doses of KP54 in healthy women and demonstrated that KP54 induced a dose-dependent (at doses from 0.2–6.4 nmol/kg) increase in LH release in all phases of the menstrual cycle, with the greatest effect in the preovulatory phase and least in the follicular phase of the cycle [50]. The ability of kisspeptin (KP54 and KP10) to stimulate gonadotropin secretion in healthy women is further confirmed in later studies [51–55]. Interestingly, intravenous injection of KP10 at doses up to 10 nmol/kg induced elevated serum gonadotropins in women during the preovulatory phase but not in women during the follicular phase of the menstrual cycle [51]. Another two studies demonstrated that KP10 stimulated LH secretion in women regardless of the menstrual cycle phase [57, 58]. One possible explanation for the inconsistency towards the effects of KP10 on women during the follicular phase is that the stimulation of KISS1R in GnRH neurons in the early follicular phase is close to saturation. Thus the treatment of additional kisspeptin has little or no effect [57]. Another explanation is that the gonadotropin response to KP10 is directly and positively related to serum estrogen levels which is much higher during the preovulatory phase than the follicular phase [54, 55]. A recent study investigated the effect of kisspeptin in young, middle, and older men and found that the response of GnRH/LH secretion to kisspeptin stimulation is preserved across life [111]. TAK-448 and TAK-683 are two KP10 analogs initially used in patients with prostate cancer [93, 99]. A recent study demonstrated that healthy women in the follicular phase treated with TAK-448 showed a similar amplitude of LH rise compared with the treatment of KP54, but the peak of LH was much later with correspondingly increased area under the curve of LH exposure [67]. The results suggest a considerable therapeutic potential of TAK-448 in female reproduction.

The change of the number of LH pulses after a bonus administration of kisspeptin in healthy women is inconsistent, with one study showing a significantly increased mean number of LH pulses [52] and another study that showed no changes [57]. While the infusion of KP54 in healthy women does not abolish the menstrual cyclicity, it leads to a reduced length of the menstrual cycle and an earlier onset LH surge [53]. Additionally, continuous infusion of KP10 restores gonadotropin pulsatility in patients with impaired GnRH secretion caused by the loss-of-function mutations in NKB or NK3R, suggesting that KP10 is sufficient to stimulate pulsatile GnRH secretion on its own [48].

Continuous exposure to GnRH or its analogs suppress gonadal functions, leading to desensitization of GnRH receptor [112, 113]. Whether long-term treatment of kisspeptin can induce tachyphylaxis in humans remains discussion. Tachyphylaxis of LH response has been seen in women with hypothalamic amenorrhea who received twice-daily subcutaneous injections of KP54 (6.4 nmol/kg) for two weeks [63, 64]. Serum LH and FSH were increased significantly after kisspeptin injection and reduced to the baseline level on day 14 [63, 64]. However, twice-daily subcutaneous injections of KP54 (6.4 nmol/kg) for one week or subcutaneous infusion of KP54 (0.1–1.0 nmol/kg/h) for 8 hours did not cause tachyphylaxis of LH response in healthy women [53, 54]. Similarly, continuous infusion of KP10 (4 μg/kg/h) for 22.5 hours in healthy men increased LH secretion progressively with no observation of desensitization [77], whereas subcutaneous infusion of TAK-448 (0.01–1 mg/man/day) or TAK-683 (0.01–2 mg/man/day) for two weeks in healthy male volunteers resulted in acute stimulation of FSH/LH followed by suppression during continuous exposure [93, 99]. These studies collectively indicate that a two-week time is required to induce tachyphylaxis in both men and women. Additionally, as tachyphylaxis is seen in women with hypothalamic amenorrhea but not healthy women, whether the status of hypothalamic amenorrhea affects tachyphylaxis remains unclear. Because chronic administration of kisspeptin is implicated as a potential novel therapy in contraception or the treatment of hormone-sensitive cancer through inhibiting reproductive hormone secretion due to tachyphylaxis, whether long-term administration of kisspeptin can induce tachyphylaxis and what is the requirement for that in humans are of considerable importance and should be confirmed in the future.

#### Trials in postmenopausal women

In postmenopausal women, the ovary cannot respond to the pituitary hormones (FSH and LH) due to the depletion of ovarian follicles. While the ovarian estrogen and progesterone production ceases and the negative feedback from the ovary is absent, the HPO axis remains intact in these women. Current evidence regarding the effect of kisspeptin on gonadotrophin secretion in postmenopausal women remains controversial. One trial demonstrated that gonadotrophin response to KP10 in these women was enhanced when compared with women in the follicular phase (George, Anderson, and Millar 2012), whereas another trial found intravenous bolus of KP10 at the same doses (0.3 μg/kg) did not affect LH or FSH secretion [56]. Therefore, the effect of kisspeptin on gonadotrophin secretion in postmenopausal women should be further confirmed.

#### Trials in women with polycystic ovary syndrome (PCOS)

PCOS is a complex disorder involved with reproductive, metabolic, and endocrine disturbances in women of reproductive age. The characteristics of PCOS include polycystic ovaries morphology, olio-anovulation, and the presence of clinical and biochemical hyperandrogenism [114]. The observation of elevated LH-pulse frequency and perturbed LH/FSH ratio in women with PCOS implied the involvement of abnormal HPO axis in PCOS [115–117]. Kisspeptin treatment leads to a similar increase of LH secretion in women with PCOS when compared with that in healthy women, and the LH pulse frequency is not changed [61, 62, 67], indicating that exogenous kisspeptin administration is not able to change the LH pulse frequency. Because NKB/NK3R pathway can promote kisspeptin secretion in the KNDy neuron, NKB antagonist administration may negatively regulate kisspeptin production and subsequently reduce GnRH/LH secretion. Indeed, previous studies demonstrated that the NK3R antagonist could reduce LH and testosterone secretion and slow LH pulse frequency in women with PCOS [62, 118]. Furthermore, LH response to kisspeptin is positively associated with estrogen concentrations [54, 55], but the positive correlation is abolished after NK3R antagonist treatment [62]. These data indicate a crucial role of the NKB component of the KNDy neuron in modulating the effect of the negative feedback of estrogen and the precise regulation of LH secretion.

#### Trials in women with hypothalamic amenorrhea (HA)

HA is characterized by the cessation of menstruation due to deficient pulsatile secretion of GnRH [119]. The absence of pulsatile GnRH secretion leads to reduced circulating gonadotropins, reduced developing follicles, low estradiol levels, and cycle disturbances [120]. Therefore, restoring pulsatile GnRH secretion is likely to be an effective treatment for HA. Gonadotrophin response to kisspeptin administration is more advanced in women with HA than in healthy women or women with PCOS [67]. Acute administration of KP54 to women with HA significantly increases gonadotrophins secretion and LH pulsatility [63–67], whereas chronic administration of KP54 at a dose of 6.4 nmol/kg twice daily for two weeks induces complete desensitization to its effects on gonadotropin release [63, 64]. In response to tachyphylaxis induced by long-term treatment of kisspeptin, Jayasena, et al. investigated the effect of administration of KP54 at a dose of 6.4 nmol/kg twice weekly for eight weeks on gonadotropin release. They demonstrated that women with HA remained partially sensitive to KP54 treatment during the first two weeks, and gonadotrophin responses to KP54 treatment did not significantly decrease beyond this initial two-week period [64]. However, this protocol of KP54 treatment could not restore menstrual cyclicity in women with HA during the eight weeks [64]. Furthermore, the number of follicles, the maximum size of the follicles, and the ovary volume were not increased in this protocol, indicating that this protocol is not therapeutic for HA. Further work is required to explore an effective protocol of kisspeptin administration that avoids tachyphylaxis and restores menstrual cyclicity and promotes the development of follicles in women with HA.

#### Trials in patients with idiopathic hypogonadotropic hypogonadism (IHH)

Idiopathic hypogonadotropic hypogonadism (IHH) is characterized by hypogonadism with low gonadotropins levels and sex steroids and absence of secondary sexual characteristics due to either pituitary or hypothalamic dysfunction [121]. As reported in previous studies, known genetic mutations account for about 30–50% of all IHH cases [122]. The majority of these genes encode proteins involved in the development and migration of GnRH neurons, regulation of GnRH secretion, and GnRH action in the pituitary [122]. GNRHR, TACR3, and KISS1R mutations are the most common mutations that lead to IHH [123, 124]. Around 10–22% of patients with IHH can recover reproductive endocrine function spontaneously [125, 126]. Exogenous pulsatile GnRH administration can serve as a powerful probe for pituitary function in IHH patients [127]. Similarly, kisspeptin, the primary upstream activator of the HPO axis, may be utilized as a potent probe for GnRH neuronal function in IHH patients. Indeed, pituitary response to GnRH is preserved in men with mutations of genes involved in the development and migration of GnRH neurons, but the pituitary response to kisspeptin is significantly attenuated when compared with healthy men (Abbara et al. 2021). Chan et al. recruited twelve patients (10 men and 2 women) with IHH (11 abiding IHH and 1 reversal IHH) who received intravenous boluses of kisspeptin (0.24 nmol/kg) before or after GnRH administration [128]. All patients with abiding IHH failed to induce LH pulse, whereas patients with reversal IHH showed robust response after exogenous kisspeptin administration. Based on this research, Lippincott et al. demonstrated that IHH patients with sustained reversal of their hypogonadotropism responded to kisspeptin and produced LH pulses, whereas IHH patients who had reversal but subsequently suffered a relapse of their IHH did not respond to kisspeptin [129]. The inability to respond to a physiologic dose of kisspeptin in IHH patients is observed in both estrogen-deficient and estrogen-replete states [130], indicating that the low estrogen level in IHH patients is not the underlying reason. This makes it possible to use kisspeptin as an adjunctive tool to assess for reversal in clinical practice. Nevertheless, not all patients with reversal IHH have a normal GnRH neuronal function because the reversal can occur even in patients with severe GnRH deficiency [126]. Therefore, these patients (reversal IHH with abnormal GnRH neuronal function) are unlikely to be accurately assessed for reversal by kisspeptin. Because most trials on IHH patients focus on men rather than women, future trials should also investigate whether the effect of kisspeptin in IHH patients shows the sexual difference.

#### Trials in women with precocious or delayed puberty

The HPO axis is active during fetal life, with a peak between mid-gestation and subsequent decrease towards the end of pregnancy [131]. A further increase in gonadotropin secretion (sometimes called the ‘mini puberty of early infancy’) is observed among human infants, beginning at 1–2 weeks after birth and persisting for several months in male infants and ∼2 years in female infants [119, 132, 133]. HPO axis remains quiescent throughout childhood and then reactivates upon the onset of puberty [131]. Reactivation of the HPO axis with the reemergence of pulsatile GnRH release is the hallmark of puberty onset [134]. Animal studies demonstrated that KISS1R expression in the arcuate nucleus region increased significantly from the juvenile to mid pubertal stage, and repeated administration of kisspeptin or its analogs was able to advance puberty [89, 135–139]. In humans, inactivating point mutations in KISS1R are associated with impaired pubertal development [13, 14]. Interestingly, mutation of the KISS1 gene that produces kisspeptins more resistant to in vitro degradation was reported in three unrelated patients with central precocious puberty [22]. These observations support the concept that the kisspeptin/KISS1R system is a therapeutic target to regulate the onset of puberty.

Central precocious puberty (CPP) is caused by the early reactivation of the HPO axis, with the development of secondary sexual characteristics before the age of 8 in girls and 9 in boys [140, 141]. In addition, several studies have shown that the level of serum kisspeptin increased significantly in CPP patients [142–146], suggesting serum kisspeptin level can be used as a potential biomarker in diagnosing CPP.

For children (11 boys and 4 girls) with delayed puberty, a wide range of responsiveness to kisspeptin administration have been reported, with some showing a robust response (higher than 0.8 mIU/ml) and others showing little to no response [147]. The neuroendocrine profiles of the responders are similar to those of healthy adults. Meanwhile, those who lacked responses to kisspeptin presented similar neuroendocrine profiles to those adults with IHH [147].. If the diminished response to kisspeptin persists into adulthood, they will be found to have IHH. Thus, response to kisspeptin may be used to distinguish physiologic hypogonadotropic hypogonadism in normal prepubertal children from the pathological hypogonadotropic hypogonadotropism of children who have IHH. Interestingly, when these children reached the age of 18 years, those who responded to kisspeptin with LH rose higher than 0.8 mIU/mL subsequently progressed through puberty, and those who showed LH responses to kisspeptin less than 0.4 mIU/ml failed to develop puberty [148]. Therefore, responses to kisspeptin can also help to predict later pubertal development in children with delayed puberty.

#### Trials in women undergoing in vitro fertilization (IVF)

IVF is a supraphysiological process that involves follicular maturation, ovulation triggering, fertilization, and embryo transfer. In this process, exogenous FSH is administered to induce the development of follicles in the ovary. Meanwhile, the GnRH receptor is downregulated by using the GnRH antagonist, or continuous treatment of a GnRH agonist to prevent an LH surge and premature ovulation. Once follicles grow to the requisite size, LH-like drugs are used to trigger ovulation [149]. Excessive exposure to LH-like drugs, such as human chorionic gonadotropin (hCG), recombinant LH (rLH), or GnRH agonist, can increase the risk of ovarian hyperstimulation syndrome (OHSS), which is an iatrogenic state that can lead to adverse consequences in women undergoing IVF treatment [150, 151]. Compared to hCG or rLH, which can directly activate the LH receptor in the ovary, GnRH agonist triggers ovulation by stimulating endogenous LH release from the pituitary, which is a more physiological process and has advantages in women with high risks of OHSS [152]. In recent years, kisspeptin has been used to induce ovulation in IVF treatment based on its ability to stimulate endogenous GnRH release and induce the subsequent LH surge. The first trial investigating kisspeptin administration to trigger ovulation was initiated in 2014 [59]. A single subcutaneous injection of KP54 was administered (1.6–12.8 nmol/kg) to induce the LH surge in women with subfertility, which resulted in a clinical pregnancy rate of 23% (12/53) and a live birth rate at 19% (10/54) [59]. Abbara et al. further explored the efficacy and safety of kisspeptin in inducing oocyte maturation in women at high risk of OHSS [153]. This study demonstrated that women treated 9.6 nmol/kg KP54 had the highest pregnancy rates (85%), and no woman developed moderate to severe OHSS [153]. The number of mature oocyte yields increased dose-dependently in both trials, indicating the variability in response in these women. A third trial was conducted to investigate whether a second dose of 9.6 nmol/kg KP54 at 10 hours following the first could result in efficacious oocyte maturation in women at high risk of OHSS [60]. This study showed the second dose of KP54 improved oocyte yield and resulted in a higher clinical pregnancy rate and live birth [60]. Notably, the rate of moderate to severe OHSS was not increased in women following a second dose of KP54 treatment. Interestingly, this study also found a pharmacodynamic effect of repeated dosing of KP54 [60]. The LH response to the second dose was likely to result from the LH response to the first KP54 bolus treatment. Women who showed a substantial LH response to the first dose had a lower LH rise following the second dose of KP54 and vice versa [60]. Therefore, the second dose of KP54 can restore and prolong the LH rise in women with a lower rise in LH following the first dose, leading to a reduced proportion of women with fewer than four oocytes yield [60]. This pharmacodynamic effect can be explained by the remaining hypothalamic GnRH stores in women with a lower rise in LH, thus allowing the second dose of KP54 to restore LH rise. In humans, hCG has a long half-life with peak serum levels at around 18 hours after injection. A peak serum LH is observed around 4 hours after injection of GnRH agonist [154] and 4 to 6 hours after injection of KP54, with a lower amplitude of LH following KP54 injection than that following GnRH agonist injection [149]. Compared to hCG and GnRH agonist for final oocyte maturation, triggered with kisspeptin leads to smaller median ovarian volume, lesser mean ascetic volumes, and least frequent OHSS symptoms in women at high risk of OHSS [155]. These data suggest that kisspeptin induces LH rise more physiologically when compared with the current protocol for oocyte maturation, particularly in women at high risks of OHSS. Importantly, injection of KP54 for final oocyte maturation is not feasible in women with an impairment in hypothalamic GnRH neuronal function. Additionally, while KP54 induces an LH surge sufficient for oocyte maturation, the duration of LH exposure is much shorter than that in hCG treatment, which may lead to the insufficient luteinizing of corpora luteal as seen in women treated with GnRH agonist [149]. Thus, adequate luteal phase support may be necessary to maintain the pregnancy rate in women triggered by KP54, and trials investigating whether the “double” or “dual” trigger by kisspeptin and hCG results in higher pregnancy rates when compared with the trigger by kisspeptin alone are of great interest.

### Kisspeptin/KISS1R outside of the hypothalamus

Emerging evidence demonstrates that the kisspeptin/KISS1R system in reproductive organs outside of the hypothalamus (such as the ovary, the endometrium, and the placenta) plays a potential role (see related reviews [12, 17]). However, the clinical application of kisspeptin based on its role in these organs is limited, probably due to the low expression of KISS1R in these organs compared with that in the hypothalamus [18]. As kisspeptin peptides are highly expressed in the placenta during pregnancy, several studies have suggested that kisspeptin measurement after six weeks of gestation of pregnant women has a potential for comparable or even higher accuracy than hCG in differentiating between miscarriage and viable intrauterine pregnancy [156–158].

### Challenges and chances

**Fig. 5:**
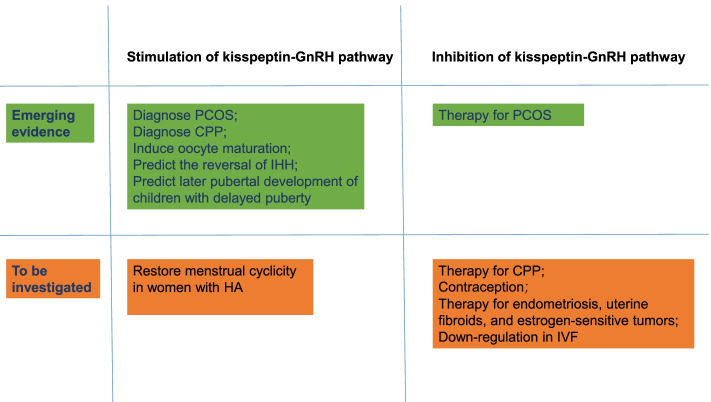
Clinical application of kisspeptin in female reproductive health. CPP, central precocious puberty; IHH, idiopathic hypogonadotropic hypogonadism; HA, hypothalamic amenorrhea; PCOS, polycystic ovary syndrome; IVF, in vitro fertilization

The discovery of the physiological role of kisspeptin/KISS1R in regulating the HPO axis has revolutionized the field of reproductive physiology since 2003 [13, 14]. Up to now, kisspeptin or its analogs have been applied in healthy women and women with abnormal conditions (IHH, HA, PCOS, abnormal puberty onset, and subfertility) (Fig. 5). Nevertheless, several unanswered questions remain to be investigated. The requirement for kisspeptin-induced tachyphylaxis and whether this tachyphylaxis shows sexual difference are not fully investigated. A commercially available pump that can be practiced internationally will enable the continuous and precise infusion of kisspeptin in some conditions. Currently, only KP54, KP10, TAK-448, and TAK-683 have been used in humans. Other promising KISS1R agonists, particularly small molecules that may facilitate oral administration, are worth investigating in future clinical trials.

Kisspeptin as the main upstream regulator of the HPO axis may have diagnostic and therapeutic significance to patients with PCOS. Accumulating studies have analyzed the serum kisspeptin level in women with PCOS, and pooled data demonstrated that kisspeptin level was significantly increased in women with PCOS and showed good accuracy for PCOS detection [159, 160]. Therefore, women with PCOS may benefit from inhibitingkisspeptin secretion in the KNDy neuron (induced by NK3R antagonist) responsible for the GnRH/LH pulse secretion [43–46]. The tachyphylaxis induced by kisspeptin allows the potential application of kisspeptin in some conditions where suppression of gonadotrophins and sex steroids is therapeutic, such as sex hormone-sensitive tumors, endometriosis, and uterine fibroids. The ability of kisspeptin antagonists to limit follicular development and inhibit ovulation may allow its potential application as a novel female contraceptive, particularly in the conditions where exogenous estrogen is contraindicated. Similar to the effect of the GnRH agonist, the ability of KISS1R agonists to induce tachyphylaxis may support their potential application in down-regulation in IVF. The use of kisspeptin and neurokinin B suppressive therapies may be used to treat CPP, and the use of kisspeptin and neurokinin B stimulating therapies may be used to restore the menstrual cyclicity in women with HA.

## Conclusion

We conducted literature reviews regarding the clinical applications of kisspeptin and/or its analogs in female reproductive health. Admittedly, the conclusive application of kisspeptin in several conditions is still pending. In addition, trials evaluating the effect of some promising KISS1R agonists are lacking. Thus, many unsolved problems exist and are waiting to be elucidated.

## Data Availability

All data generated or analysed during this study are included in this published article.

## Abbreviations

hCG: Human chorionic gonadotropin
rLH: Recombinant LH
OHSS: Ovarian hyperstimulation syndrome
CPP: Central precocious puberty
IHH: Idiopathic hypogonadotropic hypogonadism
HA: Hypothalamic amenorrhea
PCOS: Polycystic ovary syndrome
POA: Preoptic area
PeN: periventricular nucleus
AVPV: Anteroventral periventricular nucleus
HPO: Hypothalamic-pituitary-ovary
GnRH: Gonadotrophin-Releasing Hormone
FSH: Follicle-stimulating hormone
LH: Luteinizing hormone

## References

[1] Marques P, Skorupskaite K, George JT, Anderson RA, Feingold KR, Anawalt B, Boyce A, Chrousos G, de Herder WW, Dhatariya K (2000). Physiology of GNRH and Gonadotropin Secretion. Endotext.

[2] Knobil E, Plant TM, Wildt L, Belchetz PE, Marshall G (1980). Control of the rhesus monkey menstrual cycle: permissive role of hypothalamic gonadotropin-releasing hormone. Science (New York, NY).

[3] Herbison AE (1998). Multimodal influence of estrogen upon gonadotropin-releasing hormone neurons. Endocr Rev.

[4] Goodman RL (1978). The site of the positive feedback action of estradiol in the rat. Endocrinology.

[5] Herbison AE, Pape JR (2001). New evidence for estrogen receptors in gonadotropin-releasing hormone neurons. Front Neuroendocrinol.

[6] Smith JT, Cunningham MJ, Rissman EF, Clifton DK, Steiner RA (2005). Regulation of Kiss1 gene expression in the brain of the female mouse. Endocrinology.

[7] Tomikawa J, Uenoyama Y, Ozawa M, Fukanuma T, Takase K, Goto T (2012). Epigenetic regulation of Kiss1 gene expression mediating estrogen-positive feedback action in the mouse brain. Proc Natl Acad Sci U S A.

[8] Tenenbaum-Rakover Y, Commenges-Ducos M, Iovane A, Aumas C, Admoni O, de Roux N (2007). Neuroendocrine phenotype analysis in five patients with isolated hypogonadotropic hypogonadism due to a L102P inactivating mutation of GPR54. J Clin Endocrinol Metab.

[9] Roseweir AK, Millar RP (2009). The role of kisspeptin in the control of gonadotrophin secretion. Hum Reprod Update.

[10] Nimri R, Lebenthal Y, Lazar L, Chevrier L, Phillip M, Bar M (2011). A novel loss-of-function mutation in GPR54/KISS1R leads to hypogonadotropic hypogonadism in a highly consanguineous family. J Clin Endocrinol Metab.

[11] Topaloglu AK, Tello JA, Kotan LD, Ozbek MN, Yilmaz MB, Erdogan S (2012). Inactivating KISS1 mutation and hypogonadotropic hypogonadism. N Engl J Med.

[12] Hu KL, Chang HM, Zhao HC, Yu Y, Li R, Qiao J (2019). Potential roles for the kisspeptin/kisspeptin receptor system in implantation and placentation. Hum Reprod Update.

[13] de Roux N, Genin E, Carel JC, Matsuda F, Chaussain JL, Milgrom E (2003). Hypogonadotropic hypogonadism due to loss of function of the KiSS1-derived peptide receptor GPR54. Proc Natl Acad Sci U S A.

[14] Seminara SB, Messager S, Chatzidaki EE, Thresher RR, Acierno JS, Shagoury JK (2003). The GPR54 gene as a regulator of puberty. N Engl J Med.

[15] Lee JH, Miele ME, Hicks DJ, Phillips KK, Trent JM, Weissman BE (1996). KiSS-1, a novel human malignant melanoma metastasis-suppressor gene. J Natl Cancer Inst.

[16] West A, Vojta PJ, Welch DR, Weissman BE (1998). Chromosome localization and genomic structure of the KiSS-1 metastasis suppressor gene (KISS1). Genomics.

[17] Hu KL, Zhao H, Chang HM, Yu Y, Qiao J (2017). Kisspeptin/Kisspeptin receptor system in the ovary. Front Endocrinol.

[18] Lee DK, Nguyen T, O'Neill GP, Cheng R, Liu Y, Howard AD (1999). Discovery of a receptor related to the galanin receptors. FEBS Lett.

[19] Oakley AE, Clifton DK, Steiner RA (2009). Kisspeptin signaling in the brain. Endocr Rev.

[20] Herbison AE, Theodosis DT (1992). Immunocytochemical identification of oestrogen receptors in preoptic neurones containing calcitonin gene-related peptide in the male and female rat. Neuroendocrinology.

[21] Moalla M, Hadj Kacem F, Al-Mutery AF, Mahfood M, Mejdoub-Rekik N, Abid M (2019). Nonstop mutation in the Kisspeptin 1 receptor (KISS1R) gene causes normosmic congenital hypogonadotropic hypogonadism. J Assist Reprod Genet.

[22] Silveira LG, Noel SD, Silveira-Neto AP, Abreu AP, Brito VN, Santos MG (2010). Mutations of the KISS1 gene in disorders of puberty. J Clin Endocrinol Metab.

[23] Semple RK, Achermann JC, Ellery J, Farooqi IS, Karet FE, Stanhope RG (2005). Two novel missense mutations in g protein-coupled receptor 54 in a patient with hypogonadotropic hypogonadism. J Clin Endocrinol Metab.

[24] Pallais JC, Bo-Abbas Y, Pitteloud N, Crowley WF, Seminara SB (2006). Neuroendocrine, gonadal, placental, and obstetric phenotypes in patients with IHH and mutations in the G-protein coupled receptor, GPR54. Mol Cell Endocrinol.

[25] Lanfranco F, Gromoll J, von Eckardstein S, Herding EM, Nieschlag E, Simoni M (2005). Role of sequence variations of the GnRH receptor and G protein-coupled receptor 54 gene in male idiopathic hypogonadotropic hypogonadism. Eur J Endocrinol.

[26] Gottsch ML, Cunningham MJ, Smith JT, Popa SM, Acohido BV, Crowley WF (2004). A role for kisspeptins in the regulation of gonadotropin secretion in the mouse. Endocrinology.

[27] Clarkson J, Herbison AE (2006). Postnatal development of kisspeptin neurons in mouse hypothalamus; sexual dimorphism and projections to gonadotropin-releasing hormone neurons. Endocrinology..

[28] Clarkson J, d'Anglemont de Tassigny X, Colledge WH, Caraty A, Herbison AE (2009). Distribution of kisspeptin neurones in the adult female mouse brain. J Neuroendocrinol.

[29] Kauffman AS, Gottsch ML, Roa J, Byquist AC, Crown A, Clifton DK (2007). Sexual differentiation of Kiss1 gene expression in the brain of the rat. Endocrinology..

[30] Ramaswamy S, Guerriero KA, Gibbs RB, Plant TM (2008). Structural interactions between kisspeptin and GnRH neurons in the mediobasal hypothalamus of the male rhesus monkey (Macaca mulatta) as revealed by double immunofluorescence and confocal microscopy. Endocrinology..

[31] Shibata M, Friedman RL, Ramaswamy S, Plant TM (2007). Evidence that down regulation of hypothalamic KiSS-1 expression is involved in the negative feedback action of testosterone to regulate luteinising hormone secretion in the adult male rhesus monkey (Macaca mulatta). J Neuroendocrinol.

[32] Smith JT, Shahab M, Pereira A, Pau KY, Clarke IJ (2010). Hypothalamic expression of KISS1 and gonadotropin inhibitory hormone genes during the menstrual cycle of a non-human primate. Biol Reprod.

[33] Rometo AM, Krajewski SJ, Voytko ML, Rance NE (2007). Hypertrophy and increased kisspeptin gene expression in the hypothalamic infundibular nucleus of postmenopausal women and ovariectomized monkeys. J Clin Endocrinol Metab.

[34] Hrabovszky E, Ciofi P, Vida B, Horvath MC, Keller E, Caraty A (2010). The kisspeptin system of the human hypothalamus: sexual dimorphism and relationship with gonadotropin-releasing hormone and neurokinin B neurons. Eur J Neurosci.

[35] Irwig MS, Fraley GS, Smith JT, Acohido BV, Popa SM, Cunningham MJ (2004). Kisspeptin activation of gonadotropin releasing hormone neurons and regulation of KiSS-1 mRNA in the male rat. Neuroendocrinology..

[36] Matsui H, Takatsu Y, Kumano S, Matsumoto H, Ohtaki T (2004). Peripheral administration of metastin induces marked gonadotropin release and ovulation in the rat. Biochem Biophys Res Commun.

[37] Okamura H, Tsukamura H, Ohkura S, Uenoyama Y, Wakabayashi Y, Maeda K (2013). Kisspeptin and GnRH pulse generation. Adv Exp Med Biol.

[38] Herbison AE (2016). Control of puberty onset and fertility by gonadotropin-releasing hormone neurons. Nat Rev Endocrinol.

[39] Franceschini I, Lomet D, Cateau M, Delsol G, Tillet Y, Caraty A (2006). Kisspeptin immunoreactive cells of the ovine preoptic area and arcuate nucleus co-express estrogen receptor alpha. Neurosci Lett.

[40] Navarro VM, Gottsch ML, Chavkin C, Okamura H, Clifton DK, Steiner RA (2009). Regulation of gonadotropin-releasing hormone secretion by kisspeptin/dynorphin/neurokinin B neurons in the arcuate nucleus of the mouse. J Neurosci.

[41] Wakabayashi Y, Nakada T, Murata K, Ohkura S, Mogi K, Navarro VM (2010). Neurokinin B and dynorphin a in kisspeptin neurons of the arcuate nucleus participate in generation of periodic oscillation of neural activity driving pulsatile gonadotropin-releasing hormone secretion in the goat. J Neurosci.

[42] Lehman MN, Coolen LM, Goodman RL (2010). Minireview: kisspeptin/neurokinin B/dynorphin (KNDy) cells of the arcuate nucleus: a central node in the control of gonadotropin-releasing hormone secretion. Endocrinology..

[43] Navarro VM (2012). New insights into the control of pulsatile GnRH release: the role of Kiss1/neurokinin B neurons. Front Endocrinol (Lausanne).

[44] Wakabayashi Y, Yamamura T, Sakamoto K, Mori Y, Okamura H (2013). Electrophysiological and morphological evidence for synchronized GnRH pulse generator activity among Kisspeptin/neurokinin B/dynorphin a (KNDy) neurons in goats. J Reprod Develop.

[45] Ramaswamy S, Seminara SB, Ali B, Ciofi P, Amin NA, Plant TM (2010). Neurokinin B stimulates GnRH release in the male monkey (Macaca mulatta) and is colocalized with kisspeptin in the arcuate nucleus. Endocrinology..

[46] Clarkson J, Han SY, Piet R, McLennan T, Kane GM, Ng J (2017). Definition of the hypothalamic GnRH pulse generator in mice. Proc Natl Acad Sci U S A.

[47] Topaloglu AK, Reimann F, Guclu M, Yalin AS, Kotan LD, Porter KM (2009). TAC3 and TACR3 mutations in familial hypogonadotropic hypogonadism reveal a key role for neurokinin B in the central control of reproduction. Nat Genet.

[48] Young J, George JT, Tello JA, Francou B, Bouligand J, Guiochon-Mantel A (2013). Kisspeptin restores pulsatile LH secretion in patients with neurokinin B signaling deficiencies: physiological, pathophysiological and therapeutic implications. Neuroendocrinology..

[49] Skorupskaite K, George JT, Veldhuis JD, Millar RP, Anderson RA (2017). Neurokinin 3 receptor antagonism decreases gonadotropin and testosterone secretion in healthy men. Clin Endocrinol.

[50] Dhillo WS, Chaudhri OB, Thompson EL, Murphy KG, Patterson M, Ramachandran R (2007). Kisspeptin-54 stimulates gonadotropin release most potently during the preovulatory phase of the menstrual cycle in women. J Clin Endocrinol Metab.

[51] Jayasena CN, Nijher GM, Comninos AN, Abbara A, Januszewki A, Vaal ML (2011). The effects of kisspeptin-10 on reproductive hormone release show sexual dimorphism in humans. J Clin Endocrinol Metab.

[52] Jayasena CN, Comninos AN, Veldhuis JD, Misra S, Abbara A, Izzi-Engbeaya C (2013). A single injection of kisspeptin-54 temporarily increases luteinizing hormone pulsatility in healthy women. Clin Endocrinol.

[53] Jayasena CN, Comninos AN, Nijher GM, Abbara A, De Silva A, Veldhuis JD (2013). Twice-daily subcutaneous injection of kisspeptin-54 does not abolish menstrual cyclicity in healthy female volunteers. J Clin Endocrinol Metab.

[54] Narayanaswamy S, Jayasena CN, Ng N, Ratnasabapathy R, Prague JK, Papadopoulou D (2016). Subcutaneous infusion of kisspeptin-54 stimulates gonadotrophin release in women and the response correlates with basal oestradiol levels. Clin Endocrinol.

[55] Skorupskaite K, George JT, Veldhuis JD, Millar RP, Anderson RA (2016). Interactions between neurokinin B and Kisspeptin in mediating estrogen feedback in healthy women. J Clin Endocrinol Metab.

[56] Skorupskaite K, George JT, Veldhuis JD, Millar RP, Anderson RA (2018). Neurokinin 3 receptor antagonism reveals roles for neurokinin B in the regulation of gonadotropin secretion and hot flashes in postmenopausal women. Neuroendocrinology..

[57] Chan YM, Butler JP, Sidhoum VF, Pinnell NE, Seminara SB (2012). Kisspeptin administration to women: a window into endogenous kisspeptin secretion and GnRH responsiveness across the menstrual cycle. J Clin Endocrinol Metab.

[58] George JT, Anderson RA, Millar RP (2012). Kisspeptin-10 stimulation of gonadotrophin secretion in women is modulated by sex steroid feedback. Hum Reprod.

[59] Jayasena CN, Abbara A, Comninos AN, Nijher GM, Christopoulos G, Narayanaswamy S (2014). Kisspeptin-54 triggers egg maturation in women undergoing in vitro fertilization. J Clin Invest.

[60] Abbara A, Clarke S, Islam R, Prague JK, Comninos AN, Narayanaswamy S (2017). A second dose of kisspeptin-54 improves oocyte maturation in women at high risk of ovarian hyperstimulation syndrome: a phase 2 randomized controlled trial. Hum Reprod.

[61] Romero-Ruiz A, Skorupskaite K, Gaytan F, Torres E, Perdices-Lopez C, Mannaerts BM (2019). Kisspeptin treatment induces gonadotropic responses and rescues ovulation in a subset of preclinical models and women with polycystic ovary syndrome. Hum Reprod.

[62] Skorupskaite K, George JT, Veldhuis JD, Millar RP, Anderson RA (2020). Kisspeptin and neurokinin B interactions in modulating gonadotropin secretion in women with polycystic ovary syndrome. Hum Reprod.

[63] Jayasena CN, Nijher GM, Chaudhri OB, Murphy KG, Ranger A, Lim A (2009). Subcutaneous injection of kisspeptin-54 acutely stimulates gonadotropin secretion in women with hypothalamic amenorrhea, but chronic administration causes tachyphylaxis. J Clin Endocrinol Metab.

[64] Jayasena CN, Nijher GM, Abbara A, Murphy KG, Lim A, Patel D (2010). Twice-weekly administration of kisspeptin-54 for 8 weeks stimulates release of reproductive hormones in women with hypothalamic amenorrhea. Clin Pharmacol Ther.

[65] Jayasena CN, Abbara A, Veldhuis JD, Comninos AN, Ratnasabapathy R, De Silva A (2014). Increasing LH pulsatility in women with hypothalamic amenorrhoea using intravenous infusion of Kisspeptin-54. J Clin Endocrinol Metab.

[66] Millar RP, Sonigo C, Anderson RA, George J, Maione L, Brailly-Tabard S (2017). Hypothalamic-pituitary-ovarian Axis reactivation by Kisspeptin-10 in hyperprolactinemic women with chronic amenorrhea. J Endocr Soc.

[67] Abbara A, Eng PC, Phylactou M, Clarke SA, Richardson R, Sykes CM (2020). Kisspeptin receptor agonist has therapeutic potential for female reproductive disorders. J Clin Invest.

[68] García-Galiano D, Pinilla L, Tena-Sempere M (2012). Sex steroids and the control of the Kiss1 system: developmental roles and major regulatory actions. J Neuroendocrinol.

[69] Kinoshita M, Tsukamura H, Adachi S, Matsui H, Uenoyama Y, Iwata K (2005). Involvement of central metastin in the regulation of preovulatory luteinizing hormone surge and estrous cyclicity in female rats. Endocrinology..

[70] Pineda R, Garcia-Galiano D, Roseweir A, Romero M, Sanchez-Garrido MA, Ruiz-Pino F (2010). Critical roles of kisspeptins in female puberty and preovulatory gonadotropin surges as revealed by a novel antagonist. Endocrinology..

[71] de Croft S, Piet R, Mayer C, Mai O, Boehm U, Herbison AE (2012). Spontaneous kisspeptin neuron firing in the adult mouse reveals marked sex and brain region differences but no support for a direct role in negative feedback. Endocrinology..

[72] Kotani M, Detheux M, Vandenbogaerde A, Communi D, Vanderwinden JM, Le Poul E (2001). The metastasis suppressor gene KiSS-1 encodes kisspeptins, the natural ligands of the orphan G protein-coupled receptor GPR54. J Biol Chem.

[73] d'Anglemont de Tassigny X, Jayasena CN, Murphy KG, Dhillo WS, Colledge WH (2017). Mechanistic insights into the more potent effect of KP-54 compared to KP-10 in vivo. PLoS One.

[74] Jayasena CN, Abbara A, Narayanaswamy S, Comninos AN, Ratnasabapathy R, Bassett P (2015). Direct comparison of the effects of intravenous kisspeptin-10, kisspeptin-54 and GnRH on gonadotrophin secretion in healthy men. Hum Reprod.

[75] Dhillo WS, Chaudhri OB, Patterson M, Thompson EL, Murphy KG, Badman MK (2005). Kisspeptin-54 stimulates the hypothalamic-pituitary gonadal axis in human males. J Clin Endocrinol Metab.

[76] Mikkelsen JD, Bentsen AH, Ansel L, Simonneaux V, Juul A (2009). Comparison of the effects of peripherally administered kisspeptins. Regul Pept.

[77] George JT, Veldhuis JD, Roseweir AK, Newton CL, Faccenda E, Millar RP (2011). Kisspeptin-10 is a potent stimulator of LH and increases pulse frequency in men. J Clin Endocrinol Metab.

[78] Tomita K, Oishi S, Ohno H, Peiper SC, Fujii N (2008). Development of novel G-protein-coupled receptor 54 agonists with resistance to degradation by matrix metalloproteinase. J Med Chem.

[79] Niida A, Wang Z, Tomita K, Oishi S, Tamamura H, Otaka A (2006). Design and synthesis of downsized metastin (45-54) analogs with maintenance of high GPR54 agonistic activity. Bioorg Med Chem Lett.

[80] Gutiérrez-Pascual E, Leprince J, Martínez-Fuentes AJ, Ségalas-Milazzo I, Pineda R, Roa J (2009). In vivo and in vitro structure-activity relationships and structural conformation of Kisspeptin-10-related peptides. Mol Pharmacol.

[81] Tomita K, Narumi T, Niida A, Oishi S, Ohno H, Fujii N (2007). Fmoc-based solid-phase synthesis of GPR54-agonistic pentapeptide derivatives containing alkene- and fluoroalkene-dipeptide isosteres. Biopolymers..

[82] Asami T, Nishizawa N, Ishibashi Y, Nishibori K, Nakayama M, Horikoshi Y (2012). Serum stability of selected decapeptide agonists of KISS1R using pseudopeptides. Bioorg Med Chem Lett.

[83] Takino T, Koshikawa N, Miyamori H, Tanaka M, Sasaki T, Okada Y (2003). Cleavage of metastasis suppressor gene product KiSS-1 protein/metastin by matrix metalloproteinases. Oncogene..

[84] Millar RP, Newton CL (2013). Current and future applications of GnRH, kisspeptin and neurokinin B analogues. Nat Rev Endocrinol.

[85] Asami T, Nishizawa N, Ishibashi Y, Nishibori K, Horikoshi Y, Matsumoto H (2012). Trypsin resistance of a decapeptide KISS1R agonist containing an Nω-methylarginine substitution. Bioorg Med Chem Lett.

[86] Whitlock BK, Daniel JA, Amelse LL, Tanco VM, Chameroy KA, Schrick FN (2015). Kisspeptin receptor agonist (FTM080) increased plasma concentrations of luteinizing hormone in anestrous ewes. PeerJ..

[87] Curtis AE, Cooke JH, Baxter JE, Parkinson JR, Bataveljic A, Ghatei MA (2010). A kisspeptin-10 analog with greater in vivo bioactivity than kisspeptin-10. Am J Physiol Endocrinol Metab.

[88] Abbara A, Clarke SA, Dhillo WS (2021). Clinical potential of Kisspeptin in reproductive health. Trends Mol Med.

[89] Decourt C, Robert V, Anger K, Galibert M, Madinier JB, Liu X (2016). A synthetic kisspeptin analog that triggers ovulation and advances puberty. Sci Rep.

[90] Asami T, Nishizawa N, Matsui H, Nishibori K, Ishibashi Y, Horikoshi Y (2013). Design, synthesis, and biological evaluation of novel investigational nonapeptide KISS1R agonists with testosterone-suppressive activity. J Med Chem.

[91] Matsui H, Tanaka A, Yokoyama K, Takatsu Y, Ishikawa K, Asami T (2012). Chronic administration of the metastin/kisspeptin analog KISS1-305 or the investigational agent TAK-448 suppresses hypothalamic pituitary gonadal function and depletes plasma testosterone in adult male rats. Endocrinology..

[92] Matsui H, Masaki T, Akinaga Y, Kiba A, Takatsu Y, Nakata D (2014). Pharmacologic profiles of investigational kisspeptin/metastin analogues, TAK-448 and TAK-683, in adult male rats in comparison to the GnRH analogue leuprolide. Eur J Pharmacol.

[93] Scott G, Ahmad I, Howard K, MacLean D, Oliva C, Warrington S (2013). Double-blind, randomized, placebo-controlled study of safety, tolerability, pharmacokinetics and pharmacodynamics of TAK-683, an investigational metastin analogue in healthy men. Br J Clin Pharmacol.

[94] Liu X, Herbison AE (2014). RF9 excitation of GnRH neurons is dependent upon Kiss1r in the adult male and female mouse. Endocrinology..

[95] Min L, Leon S, Li H, Pinilla L, Carroll RS, Tena-Sempere M (2015). RF9 acts as a KISS1R agonist in vivo and in vitro. Endocrinology..

[96] Doebelin C, Bertin I, Schneider S, Schmitt M, Bourguignon JJ, Ancel C (2016). Development of Dipeptidic hGPR54 agonists. Chem Med Chem..

[97] Parker PA, Coffman EA, Pohler KG, Daniel JA, Aucagne V, Beltramo M (2019). Acute and subacute effects of a synthetic kisspeptin analog, C6, on serum concentrations of luteinizing hormone, follicle stimulating hormone, and testosterone in prepubertal bull calves. Theriogenology..

[98] Nishizawa N, Takatsu Y, Kumano S, Kiba A, Ban J, Tsutsumi S (2016). Design and synthesis of an investigational Nonapeptide KISS1 receptor (KISS1R) agonist, ac-d-Tyr-Hydroxyproline (Hyp)-Asn-Thr-Phe-azaGly-Leu-Arg (me)-Trp-NH(2) (TAK-448), with highly potent testosterone-suppressive activity and excellent water solubility. J Med Chem.

[99] MacLean DB, Matsui H, Suri A, Neuwirth R, Colombel M (2014). Sustained exposure to the investigational Kisspeptin analog, TAK-448, down-regulates testosterone into the castration range in healthy males and in patients with prostate cancer: results from two phase 1 studies. J Clin Endocrinol Metab.

[100] Tanaka A, Nakata D, Masaki T, Kusaka M, Watanabe T, Matsui H (2018). Evaluation of pharmacokinetics/pharmacodynamics and efficacy of one-month depots of TAK-448 and TAK-683, investigational kisspeptin analogs, in male rats and an androgen-dependent prostate cancer model. Eur J Pharmacol.

[101] Orsini MJ, Klein MA, Beavers MP, Connolly PJ, Middleton SA, Mayo KH (2007). Metastin (KiSS-1) mimetics identified from peptide structure-activity relationship-derived pharmacophores and directed small molecule database screening. J Med Chem.

[102] Pineda R, Garcia-Galiano D, Sanchez-Garrido MA, Romero M, Ruiz-Pino F, Aguilar E (2010). Characterization of the potent gonadotropin-releasing activity of RF9, a selective antagonist of RF-amide-related peptides and neuropeptide FF receptors: physiological and pharmacological implications. Endocrinology..

[103] Simonin F, Schmitt M, Laulin JP, Laboureyras E, Jhamandas JH, MacTavish D (2006). RF9, a potent and selective neuropeptide FF receptor antagonist, prevents opioid-induced tolerance associated with hyperalgesia. Proc Natl Acad Sci U S A.

[104] Roseweir AK, Millar RP (2013). Kisspeptin antagonists. Adv Exp Med Biol.

[105] Roseweir AK, Kauffman AS, Smith JT, Guerriero KA, Morgan K, Pielecka-Fortuna J (2009). Discovery of potent kisspeptin antagonists delineate physiological mechanisms of gonadotropin regulation. J Neurosci.

[106] Albers-Wolthers CHJ, de Gier J, Walen M, van Kooten PJS, Lambalk CB, Leegwater PAJ (2017). In vitro and in vivo effects of kisspeptin antagonists p234, p271, p354, and p356 on GPR54 activation. PLoS One.

[107] Smith JT, Li Q, Yap KS, Shahab M, Roseweir AK, Millar RP (2011). Kisspeptin is essential for the full preovulatory LH surge and stimulates GnRH release from the isolated ovine median eminence. Endocrinology..

[108] Kobayashi T, Sasaki S, Tomita N, Fukui S, Kuroda N, Nakayama M (2010). Synthesis and structure-activity relationships of 2-acylamino-4,6-diphenylpyridine derivatives as novel antagonists of GPR54. Bioorg Med Chem.

[109] Kobayashi T, Sasaki S, Tomita N, Fukui S, Nakayama M, Kiba A (2010). 2-acylamino-4,6-diphenylpyridine derivatives as novel GPR54 antagonists with good brain exposure and in vivo efficacy for plasma LH level in male rats. Bioorg Med Chem.

[110] Jayasena CN, Comninos AN, Narayanaswamy S, Bhalla S, Abbara A, Ganiyu-Dada Z (2014). Acute and chronic effects of kisspeptin-54 administration on GH, prolactin and TSH secretion in healthy women. Clin Endocrinol.

[111] Ullah H, Nabi G, Zubair H, Ullah R, Shahab M (2019). Age-dependent changes in the reproductive axis responsiveness to kisspeptin-10 administration in healthy men. Andrologia..

[112] Belchetz PE, Plant TM, Nakai Y, Keogh EJ, Knobil E (1978). Hypophysial responses to continuous and intermittent delivery of hypopthalamic gonadotropin-releasing hormone. Science..

[113] Millar RP, Lu ZL, Pawson AJ, Flanagan CA, Morgan K, Maudsley SR (2004). Gonadotropin-releasing hormone receptors. Endocr Rev.

[114] Rosenfield RL, Ehrmann DA (2016). The pathogenesis of polycystic ovary syndrome (PCOS): the hypothesis of PCOS as functional ovarian Hyperandrogenism revisited. Endocr Rev.

[115] Panidis D, Farmakiotis D, Rousso D, Katsikis I, Kourtis A, Diamanti-Kandarakis E (2005). Serum luteinizing hormone levels are markedly increased and significantly correlated with Delta 4-androstenedione levels in lean women with polycystic ovary syndrome. Fertil Steril.

[116] Jayasena CN, Franks S (2014). The management of patients with polycystic ovary syndrome. Nat Rev Endocrinol.

[117] Burt Solorzano CM, Beller JP, Abshire MY, Collins JS, McCartney CR, Marshall JC (2012). Neuroendocrine dysfunction in polycystic ovary syndrome. Steroids..

[118] George JT, Kakkar R, Marshall J, Scott ML, Finkelman RD, Ho TW (2016). Neurokinin B receptor antagonism in women with polycystic ovary syndrome: a randomized, placebo-controlled trial. J Clin Endocrinol Metab.

[119] Yen SS (1993). Female hypogonadotropic hypogonadism. Hypothalamic amenorrhea syndrome. Endocrinol Metab Clin N Am.

[120] Berga SL, Girton LG (1989). The psychoneuroendocrinology of functional hypothalamic amenorrhea. Psychiatr Clin North Am.

[121] Hoffman AR, Crowley WF (1982). Induction of puberty in men by long-term pulsatile administration of low-dose gonadotropin-releasing hormone. N Engl J Med.

[122] Bianco SD, Kaiser UB (2009). The genetic and molecular basis of idiopathic hypogonadotropic hypogonadism. Nat Rev Endocrinol.

[123] Gürbüz F, Kotan LD, Mengen E, Şıklar Z, Berberoğlu M, Dökmetaş S (2012). Distribution of gene mutations associated with familial normosmic idiopathic hypogonadotropic hypogonadism. J Clin Res Pediatr Endocrinol.

[124] Francou B, Paul C, Amazit L, Cartes A, Bouvattier C, Albarel F (2016). Prevalence of KISS1 receptor mutations in a series of 603 patients with normosmic congenital hypogonadotrophic hypogonadism and characterization of novel mutations: a single-Centre study. Hum Reprod.

[125] Raivio T, Falardeau J, Dwyer A, Quinton R, Hayes FJ, Hughes VA (2007). Reversal of idiopathic hypogonadotropic hypogonadism. N Engl J Med.

[126] Sidhoum VF, Chan YM, Lippincott MF, Balasubramanian R, Quinton R, Plummer L (2014). Reversal and relapse of hypogonadotropic hypogonadism: resilience and fragility of the reproductive neuroendocrine system. J Clin Endocrinol Metab.

[127] Sheckter CB, McLachlan RI, Tenover JS, Matsumoto AM, Burger HG, de Kretser DM (1988). Stimulation of serum inhibin concentrations by gonadotropin-releasing hormone in men with idiopathic hypogonadotropic hypogonadism. J Clin Endocrinol Metab.

[128] Chan YM, Lippincott MF, Butler JP, Sidhoum VF, Li CX, Plummer L (2014). Exogenous kisspeptin administration as a probe of GnRH neuronal function in patients with idiopathic hypogonadotropic hypogonadism. J Clin Endocrinol Metab.

[129] Lippincott MF, Chan YM, Delaney A, Rivera-Morales D, Butler JP, Seminara SB (2016). Kisspeptin responsiveness signals emergence of reproductive endocrine activity: implications for human puberty. J Clin Endocrinol Metab.

[130] Lippincott MF, Nguyen K, Delaney A, Chan YM, Seminara SB (2018). Assessing sex steroid influence on Kisspeptin responsiveness in idiopathic hypogonadotropic hypogonadism. J Endocr Soc.

[131] Spaziani M, Tarantino C, Tahani N, Gianfrilli D, Sbardella E, Lenzi A (2021). Hypothalamo-pituitary axis and puberty. Mol Cell Endocrinol.

[132] Abbara A, Eng PC, Phylactou M, Clarke SA, Mills E, Chia G (2020). Kisspeptin-54 accurately identifies hypothalamic GnRH neuronal dysfunction in men with congenital hypogonadotropic hypogonadism. Neuroendocrinology..

[133] Laughlin GA, Dominguez CE, Yen SS (1998). Nutritional and endocrine-metabolic aberrations in women with functional hypothalamic amenorrhea. J Clin Endocrinol Metab.

[134] Palmert MR, Boepple PA (2001). Variation in the timing of puberty: clinical spectrum and genetic investigation. J Clin Endocrinol Metab.

[135] Shahab M, Mastronardi C, Seminara SB, Crowley WF, Ojeda SR, Plant TM (2005). Increased hypothalamic GPR54 signaling: a potential mechanism for initiation of puberty in primates. Proc Natl Acad Sci U S A.

[136] Vazquez MJ, Toro CA, Castellano JM, Ruiz-Pino F, Roa J, Beiroa D (2018). SIRT1 mediates obesity- and nutrient-dependent perturbation of pubertal timing by epigenetically controlling Kiss1 expression. Nat Commun.

[137] Plant TM, Ramaswamy S, Dipietro MJ (2006). Repetitive activation of hypothalamic G protein-coupled receptor 54 with intravenous pulses of kisspeptin in the juvenile monkey (Macaca mulatta) elicits a sustained train of gonadotropin-releasing hormone discharges. Endocrinology..

[138] Navarro VM, Fernández-Fernández R, Castellano JM, Roa J, Mayen A, Barreiro ML (2004). Advanced vaginal opening and precocious activation of the reproductive axis by KiSS-1 peptide, the endogenous ligand of GPR54. J Physiol.

[139] Keen KL, Wegner FH, Bloom SR, Ghatei MA, Terasawa E (2008). An increase in kisspeptin-54 release occurs with the pubertal increase in luteinizing hormone-releasing hormone-1 release in the stalk-median eminence of female rhesus monkeys in vivo. Endocrinology..

[140] Carel JC, Léger J (2008). Clinical practice. Precocious puberty. N Engl J Med.

[141] Boehm U, Bouloux PM, Dattani MT, de Roux N, Dodé C, Dunkel L (2015). Expert consensus document: European Consensus Statement on congenital hypogonadotropic hypogonadism--pathogenesis, diagnosis and treatment. Nat Rev Endocrinol.

[142] Li M, Chen Y, Liao B, Tang J, Zhong J, Lan D (2021). The role of kisspeptin and MKRN3 in the diagnosis of central precocious puberty in girls. Endocr Connect.

[143] de Vries L, Shtaif B, Phillip M, Gat-Yablonski G (2009). Kisspeptin serum levels in girls with central precocious puberty. Clin Endocrinol.

[144] Demirbilek H, Gonc EN, Ozon A, Alikasifoglu A, Kandemir N (2012). Evaluation of serum kisspeptin levels in girls in the diagnosis of central precocious puberty and in the assessment of pubertal suppression. J Pediatr Endocrinol Metab.

[145] Rhie YJ, Lee KH, Eun SH, Choi BM, Chae HW, Kwon AR (2011). Serum kisspeptin levels in Korean girls with central precocious puberty. J Korean Med Sci.

[146] Özgen İT, Torun E, Bayraktar-Tanyeri B, Durmaz E, Kılıç E, Cesur Y (2016). The relation of urinary bisphenol a with kisspeptin in girls diagnosed with central precocious puberty and premature thelarche. J Pediatr Endocrinol Metab.

[147] Chan YM, Lippincott MF, Kusa TO, Seminara SB (2018). Divergent responses to kisspeptin in children with delayed puberty. JCI Insight.

[148] Chan YM, Lippincott MF, Sales Barroso P, Alleyn C, Brodsky J, Granados H (2020). Using Kisspeptin to predict pubertal outcomes for youth with pubertal delay. J Clin Endocrinol Metab.

[149] Abbara A, Clarke SA, Dhillo WS (2018). Novel concepts for inducing final oocyte maturation in in vitro fertilization treatment. Endocr Rev.

[150] Humaidan P, Nelson SM, Devroey P, Coddington CC, Schwartz LB, Gordon K (2016). Ovarian hyperstimulation syndrome: review and new classification criteria for reporting in clinical trials. Hum Reprod.

[151] Nastri CO, Teixeira DM, Moroni RM, Leitão VM, Martins WP (2015). Ovarian hyperstimulation syndrome: pathophysiology, staging, prediction and prevention. Ultrasound Obstet Gynecol.

[152] Humaidan P, Papanikolaou EG, Tarlatzis BC (2009). GnRHa to trigger final oocyte maturation: a time to reconsider. Hum Reprod.

[153] Abbara A, Jayasena CN, Christopoulos G, Narayanaswamy S, Izzi-Engbeaya C, Nijher GM (2015). Efficacy of Kisspeptin-54 to trigger oocyte maturation in women at high risk of ovarian Hyperstimulation syndrome (OHSS) during in vitro fertilization (IVF) therapy. J Clin Endocrinol Metab.

[154] Vuong TN, Ho MT, Ha TD, Phung HT, Huynh GB, Humaidan P (2016). Gonadotropin-releasing hormone agonist trigger in oocyte donors co-treated with a gonadotropin-releasing hormone antagonist: a dose-finding study. Fertil Steril.

[155] Abbara A, Islam R, Clarke SA, Jeffers L, Christopoulos G, Comninos AN (2018). Clinical parameters of ovarian hyperstimulation syndrome following different hormonal triggers of oocyte maturation in IVF treatment. Clin Endocrinol.

[156] Li R, Hu KL (2021). Kisspeptin, a promising biomarker for miscarriage in early pregnancy. Fertil Steril.

[157] Abbara A, Al-Memar M, Phylactou M, Kyriacou C, Eng PC, Nadir R (2021). Performance of plasma kisspeptin as a biomarker for miscarriage improves with gestational age during the first trimester. Fertil Steril.

[158] Sullivan-Pyke C, Haisenleder DJ, Senapati S, Nicolais O, Eisenberg E, Sammel MD (2018). Kisspeptin as a new serum biomarker to discriminate miscarriage from viable intrauterine pregnancy. Fertil Steril.

[159] Varikasuvu SR, Prasad VS, Vamshika VC, Satyanarayana MV, Panga JR (2019). Circulatory metastin/kisspeptin-1 in polycystic ovary syndrome: a systematic review and meta-analysis with diagnostic test accuracy. Reprod BioMed Online.

[160] de Assis Rodrigues NP, Laganà AS, Zaia V, Vitagliano A, Barbosa CP, de Oliveira R (2019). The role of Kisspeptin levels in polycystic ovary syndrome: a systematic review and meta-analysis. Arch Gynecol Obstet.

